# Unsupervised but not supervised gait parameters are related to fatigue in Parkinson’s disease: a pilot study

**DOI:** 10.3389/fnagi.2023.1279722

**Published:** 2023-11-21

**Authors:** Andrea Pilotto, Andrea Rizzardi, Cinzia Zatti, Clint Hansen, Antonio Donini, Robbin Romijnders, Walter Maetzler, Alessandro Padovani

**Affiliations:** ^1^Neurology Unit, Department of Clinical and Experimental Sciences, University of Brescia, Brescia, Italy; ^2^Laboratory of Digital Neurology and Biosensors, University of Brescia, Brescia, Italy; ^3^Neurology Unit, Department of Continuity of Care and Frailty, ASST Spedali Civili Brescia Hospital, Brescia, Italy; ^4^Department of Neurology, University Hospital Schleswig-Holstein, Kiel, Germany; ^5^Department of Neurology, Kiel University, Kiel, Germany

**Keywords:** gait analysis, fatigue, Parkinson’s disease, supervised and unsupervised assessment, mobile health technology

## Abstract

**Introduction:**

Fatigue is a common and disabling symptom in Parkinson’s disease (PD), also affecting gait. Detection of fatigue-associated changes of gait using mobile health technologies (MHT) could become increasingly effective.

**Methods:**

Cognitively unimpaired PD patients without fluctuations (UPDRS IV < 1) underwent a standard neurological assessment including the PD-Fatigue scale (PFS-16). PD patients with (PD-F) and without fatigue (PD-N) were matched for age, sex, cognitive function and disease severity. Each participant underwent MHT gait assessment under supervised condition (SC) and unsupervised condition (UC).

**Results:**

Gait parameters of 21 PD-F and 21 PD-N did not significantly differ under SC. Under UC, PD-F showed higher step time, step time variability and asymmetry index compared to PD-N and the PFS-16 correlated with step time.

**Conclusion:**

This is the first MHT-based study with PD patients showing a correlation between fatigue and gait parameters. In addition, the data collected suggest that UC is clearly superior to SC in addressing this question.

## Introduction

Fatigue is a common and disabling symptom in Parkinson’s disease (PD) (Jenkinson et al., 1999; Barone et al., 2009; Gallagher et al., 2010) affecting between 30 and 70 percent of PD patients (Siciliano et al., 2018). This makes fatigue an important target for pharmacological and non-pharmacological interventions (Friedman et al., 2010; Stocchi et al., 2014). The dramatic impact fatigue on quality of life (van Uem et al., 2016) may be mediated by various PD-related symptoms, with movement and mobility deficits playing a central role. For example, it was shown that, in PD, fatigue is associated with the Postural Instability/Gait Difficulty (PIGD) subtype (Hagell and Brundin, 2009; Zhou et al., 2023) and higher total motor sores (Hagell and Brundin, 2009; Zhou et al., 2023). In the general population, fatigue can have a significant impact on mobility and particularly on gait parameters, namely a decrease of walking speed and step length and an increase of step variability (Barbieri et al., 2013; Hamacher et al., 2016). In PD, however, the relationship between fatigue and gait-related parameters has never been explored using digital assessment and thus represent a still debated issue in clinical research (Rochester et al., 2006).

Mobile health technologies (MHT) enable the collection of quantitative and qualitative aspects of gait with high accuracy both in the professional clinical-scientific environment (supervised condition, SC) and in the home of users or patients (unsupervised condition, UC). Several studies suggest that assessment under SC, but not under UC, underestimate PD-related gait deficits (Del Din et al., 2016b; Warmerdam et al., 2020). This might especially true for fluctuating symptoms particularly sensitive to motivation and alertness such as fatigue (Jenkinson et al., 1999; Rochester et al., 2006; Barone et al., 2009; Gallagher et al., 2010).

We therefore investigated in this pilot prospective observational study gait changes in PD patients with and without fatigue matched for age, sex, motor, and cognitive severity. Aim of the study was to evaluate the relationship between gait parameters and fatigue using MHT, with the hypothesis that an home unsupervised assessment might be more informative compared to supervised clinical evaluation in showing this association.

## Methods

### Participants and clinical assessment

A total of 80 patients with a clinically established PD (Postuma et al., 2015) with at least 1 year of follow-up after diagnosis and beneficial response to dopaminergic treatment from the outpatient Movement disorder Clinic, Neurology Unit at the University of Brescia, Italy from March 2018 to December 2022. The research protocol was approved by the Ethics Committee of the Brescia Hospital, Brescia, Italy (DMA study, NP 1471/22). Written informed consent was obtained from all participants.

Only patients under stable dopaminergic treatment (Postuma et al., 2015) were included. Exclusion criteria were features suggesting atypical parkinsonism (Postuma et al., 2015), a diagnosis of dementia (Emre et al., 2007), impulse control disorder, symptomatic orthostatic hypotension, motor fluctuations (UPDRS-IV > 1), other neurologic disorders or medical conditions potentially affecting gait, need of walking aids, major depressive and bipolar disorder needing pharmacological treatment, schizophrenia, and history of drug and alcohol abuse.

All patients underwent a neurological examination, including the Movement Disorder Society-Unified Parkinson Disease Rating Scale (MDS-UPDRS) part III and IV (Goetz et al., 2008), the Hoehn and Yahr (H&Y) staging (Goetz et al., 2004) and a global cognitive screening using the Montreal Cognitive Assessment test (MOCA) (Nasreddine et al., 2005). Fatigue was assessed using the PD Fatigue Scale (PFS-16) (Brown et al., 2005). Twenty-one patients had ≥ 8 points on the PFS-16 and were considered as the PD group with fatigue (PD-F) (Friedman et al., 2010). Twenty-one PD patients without fatigue (PFS16 < 8, PD-N) matched for age, sex, cognitive function and disease severity were also included.

### Gait assessment with mobile health technologies and data extraction

This selected 42 patients underwent gait assessment under SC and UC (Supplementary Figure 1). Gait assessment under SC was performed with a RehaGait^®^ device on the lower back (Hasomed GmbH, Magdeburg, Germany) (Geritz et al., 2020). Participants walked a 20 m walkway up and down first with self-selected, then with fast speed (Lee et al., 2005). Gait assessment under UC was performed with a Move IV^®^ device on the lower back (Movisens GmbH, Karlsruhe, Germany) over four consecutive days (Härtel et al., 2011). Patients were randomly assigned to start the evaluation with either supervised or unsupervised assessment.

Inertial sensors used in the study for supervised and unsupervised data evaluate the same gait parameters (all included in the analyses) based on lower back algorithms, as extensively described in the ComON study design and in the next session (Geritz et al., 2020).

### Data processing and extracted parameters

Raw data were processed using Matlab R2022b (MathWorks, Natick, MA, USA). To analyze gait parameters the raw data of the IMU from the lower back was used for both supervised and unsupervised assessments. To ensure no systematic biases between assessments in terms of heterogeneity, usage and accuracy, raw data of accelerometer and gyroscope from the two assessments were processed with the same validated algorithm (Del Din et al., 2016a; Pham et al., 2017).

As described in the reference (Pham et al., 2017), the raw accelerometer and gyroscope data were processed to first detect the gait events. From the home assessment mean gait parameters per walking bout (WB) was calculated. WBs were determined with more than 3 consecutive steps and then were considered for further analysis. UC gait bouts were split into short (4–19 steps) and long gait bouts (≥ 20 steps) based on the number of steps (Riva et al., 2014; Rennie et al., 2018; Shah et al., 2020) for each day of recording.

### Statistical analysis

Statistical analyses were performed using IBM SPSS statistics version 26. Comparisons of clinical and demographic parameters between PD-F and PD-N were done with Mann-Whitney-U and Fisher’s exact test. Step time, step time variability and step time asymmetry (Paraschiv-Ionescu et al., 2019) were then compared between groups in SC and UC, using Mann Whitney non-parametric analysis and adjusting for age, sex, height, and disease duration. Correlations between PFS-16 values and gait parameters were done using partial correlation analyses and again adjusting for age, sex, height, and disease duration. *P* < 0.05 was considered statistically significant.

## Results

The PD-F group did not significantly differ from the PD-N group in term of age, disease duration, gender distribution, and MDS-UPDRS-III, MoCA, and BMI scores, respectively (Table 1). Under SC, none of the investigated gait parameters did significantly differ between PD-F and PD-N, neither during self-selected, nor during fast pace. Moreover, gait parameters from the SC of the entire PD cohort did not significantly correlate with any demographic or clinical parameter.

**TABLE 1:** Clinical characteristics of the patients.

	PD-N	PD-F	*p*-value
Participants (n)	21	21	
Age (years)	64.9 ± 8.0	69.6 ± 7.5	0.061^a^
Sex (% male)	71.4	52.4	0.204^b^
Disease duration (years)	5.5 ± 3.1	4.6 ± 2.8	0.230^a^
MDS-UPDRS-III (0–132)	12.8 ± 5.6	15.9 ± 9.5	0.378^a^
MDS UPDRS-III gait subscore (% > 1 score)	38.1	42.9	0.753^b^
MDS UPDRS-III tremor subscore (% > 1 score)	33.3	47.6	0.217^b^
MDS UPDRS-III rigidity subscore (% > 1 score)	71.4	66.6	0.683^b^
Height (m)	1.70 ± 0.10	1.67 ± 0.08	0.287^a^
MoCA Score (0–30)	27.4 ± 2.1	26.9 ± 2.1	0.646^a^
LEDD	370 ± 209	345 ± 212	0.693^a^
L-dopa LEDD	275 ± 181	305 ± 189	0.533^a^
DA-LEDD	53.4 ± 62.0	47.1 ± 55.5	0.725^a^
PFS-16 Score (0–16)	2.1 ± 2.2	9.3 ± 2.1	**±0.001^a^**

*^a^*Mann-Whitney-U test, *^b^*Fisher’s exact test. BMI, body mass index; DA-LEDD, dopamine-agonist LEDD; MoCA, Montreal Cognitive Assessment; L-dopa-LEDD, Levodopa LEDD; LEDD, levodopa equivalent daily dose; PFS-16, Parkinson’s disease fatigue score. Bold values represent *p* < 0.05.

Under UC, PD-F showed higher step time (*p* = 0.02), step time variability (*p* = 0.005) and step time asymmetry (*p* = 0.01), compared to PD-N. These differences were more pronounced in the long gait bouts, and less pronounced in the short gait bouts. Details are provided in Table 2. No correlation between digital parameters and PFS-16 scores were found in the whole group. In PD-F, PFS-16 total score correlated with the step time in the longer (*r* = 0.46, *p* = 0.05) and shorter bouts (*r* = 0.56, *p* = 0.013), whereas no correlation was observed in SC.

**TABLE 2:** Differences in gait parameters between PD with and without fatigue in supervised and unsupervised conditions.

Gait parameters	PD-N	PD-F	*p*-value ^c^
**Supervised assessment**			
**Straight walk self-selected pace**			
Steps (N)	113 (88–131)	108 (96–119)	0.273^c^
Step time (s)	0.53 (0.45–0.67)	0.55 (0.49–0.70)	0.116^c^
Step time variability (s)	0.04 (0.02–0.10)	0.05 (0.03–0.12)	0.264^c^
Step time asymmetry	0.02 (0.00–0.04)	0.03 (0.00–0.08)	0.125^c^
**Straight walk fast pace**			
Steps (N)	118 (102–159)	123 (110–159)	0.320^c^
Step time (s)	0.46 (0.38–0.57)	0.49 (0.38–0.54)	0.108^c^
Step time variability (s)	0.04 (0.02–0.09)	0.06 (0.02–0.09)	0.140^c^
Step time asymmetry	0.01 (0.00–0.04)	0.02 (0.00–0.05)	0.207^c^
**Unsupervised assessment**			
**Daily Bout**			
Steps (N)	31 (11.1–83.5)	30 (14.7–74.3)	0.125^d^
Step time (s)	0.74 (0.66–0.78)	0.78 (0.69–0.90)	**0.007^d^**
Step time variability (s)	0.24 (0.17–0.39)	0.29 (0.23–0.37)	**0.004^d^**
Step time asymmetry	0.11 (0.07–0.21)	0.13 (0.09–0.20)	**0.004^d^**
**Gait bouts < 20 steps**			
Steps (N)	14 (8.5–16.2)	15 (8.0–16.0)	0.269^d^
Step time (s)	0.78 (0.72–0.87)	0.83 (0.70 ± 0.99)	**0.017^d^**
Step time variability (s)	0.31 (0.18–0.40)	0.34 (0.28–0.43)	**0.017^d^**
Step time asymmetry	0.16 (0.09–0.23)	0.17 (0.11–0.24)	**0.032^d^**
**Gait bouts ≥ 20 steps**			
Steps (N)	52 (32–167)	50 (30–153)	0.250^d^
Step time (s)	0.67 (0.61–0.73)	0.69 (0.65–0.80)	**0.013^d^**
Step time variability (s)	0.19 (0.15–0.34)	0.23 (0.18–0.30)	**0.002^d^**
Step time asymmetry	0.06 (0.04–0.08)	0.07 (0.05–0.09)	**0.010^d^**
**Longest bout**			
Steps (N)	892 (58–3,089)	500 (56–2,476)	0.175^d^
Step time (s)	0.55 (0.46–0.69)	0.59 (0.53–0.88)	**0.009^d^**
Step time variability (s)	0.05 (0.02–0.21)	0.08 (0.05–0.36)	**0.029^d^**
Step time asymmetry	0.01 (0.00–0.03)	0.02 (0.00–0.06)	**0.005^d^**

Values have been indicated as mean (25th–75th percentile).

*^c^*Comparison between PD-N and PD-F have been performed using non-parametric multivariate analyses adjusted for the effect of age, sex, height, disease duration, and number of steps.

*^d^*Comparison between PD-N and PD-F have been performed using non-parametric multivariate analyses adjusted for the effect of age, sex, height, disease duration, and number of steps. Bold values represent *p* < 0.05.

## Discussion

This pilot study addressed the complex relationship between fatigue and gait changes in PD patients under supervised and unsupervised home assessment. The findings showed, to our best knowledge for the first time, that subjects with higher fatigue levels present worse gait parameters compared with subjects not reporting fatigue but only when measured in UC using MHT. Conversely, gait parameters as collected in the clinic did not reveal any significant difference between PD patients with and without fatigue. These novel results are of high interest for the research community, as the association between fatigue and “measurable” mobility parameter is still a debated issue in research.

Compared to PD-N, PD-F indeed exhibited in the home environment longer step time and increased step time variability and asymmetry index, which are the typical PD-associated features (Mirelman et al., 2019). Longer step time and step time asymmetry indicate more severe gait alterations in PD-F, whereas increased gait variability has been consistently associated with an increased risk of accelerated progression and an increased risk of falls in PD (Schaafsma et al., 2003).

The study design allowed a matching of PD with similar disease duration and severity, dopaminergic treatment and cognitive function excluding patients with depression or motor fluctuations, in order to minimize the variables potentially influencing both fatigue and motor performances at home and in the clinic (Stocchi et al., 2014; Del Din et al., 2016b; Siciliano et al., 2018; Corrà et al., 2021; Denk et al., 2022; Marin et al., 2022). The differences observed between supervised and unsupervised environment are probably due to different factors, including alertness, motivation, the white-coat and the reverse white-coat effects (respectively worsening and improvement of a clinical parameter measured in clinical setting) and the Hawthorne effect (i.e., change in participants’ behavior due to the awareness that they are being studied) (Warmerdam et al., 2020). In fact, these factors may mask fatigue aspect, and argue for an easier and more consistent role for unsupervised home evaluation for such type of target-variable and assessments.

Of interest, the differences observed in step time and step time variability between the groups in UC were, at least upon visual inspection, more prominent in long walking bouts that are more comparable to supervised analyses (Del Din et al., 2016a). Longer bouts may also reflect a more automatic walking conditions with less alertness and thus higher risk of both fatigue and classical dopamine-related features.

Several further study limitations should be acknowledged. First, the sample size and the pilot nature of this study did not allow specific sex- or fatigue-subcategory analyses (i.e., mental vs. physical), that need to be verified in larger studies. Second, the cross-sectional study design cannot evaluate changes in fatigue and mobility over time. Third, this study excluded patients with major depression, but the link between subtle depressive symptoms psychosocial wellbeing and fatigue should be definitively further explored in larger samples. Fourth, the study excluded *a priori* PD patients with motor fluctuations to limit the variability of both fatigue and mobility. Fatigue, however, is known to represent an important symptom of L-dopa-induced fluctuation and further studies focused on this specific population are pivotal also to challenge new pharmacological approaches.

Limitations notwithstanding, this study shows that fatigue in PD is associated with specific gait changes which are detectable in home-based environment using MHT technology.

These novel findings can be further investigated in ongoing studies, such as the large IMI consortium IDEA-FAST^1^ including home-based diaries and longer unsupervised evaluation, which will enable a deeper understanding of the complex relationship between fatigue, mobility and daily activities.

## Data availability statement

The raw data supporting the conclusions of this article will be made available by the authors, without undue reservation.

## Ethics statement

The studies involving humans were approved by the Ethics Committee of the Brescia Hospital, Brescia, Italy (DMA study, NP 1471/22). The studies were conducted in accordance with the local legislation and institutional requirements. The participants provided their written informed consent to participate in this study.

## Author contributions

APi: Conceptualization, Formal analysis, Methodology, Writing—original draft, Writing—review and editing. AR: Data curation, Formal analysis, Writing—original draft. CZ: Writing—review and editing. CH: Data curation, Formal analysis, Software, Writing—review and editing. AD: Data curation, Formal analysis, Software, Writing—review and editing. RR: Data curation, Formal analysis, Software, Writing—review and editing. WM: Conceptualization, Supervision, Validation, Writing—review and editing. APa: Conceptualization, Supervision, Validation, Writing—review and editing.

## Footnotes

1
https://idea-fast.eu
