# Supplementary material for: Unsupervised but not supervised gait parameters are related to fatigue in Parkinson’s disease: a pilot study
Source: Front Aging Neurosci. 2023 Nov 21;15:1279722. doi: 10.3389/fnagi.2023.1279722 (PMC10702762; doi:10.3389/fnagi.2023.1279722)
Supplement: Supplementary file 1 [file Data_Sheet_1.docx]

**Supplementary material**

**Technical details**

**Supervised assessment**

The RehaGait® system consists of three mobile inertial sensors (dimensions: 60 × 15 × 35 mm); each sensor comprises a 3-axis accelerometer (±16 g), a 3- axis gyroscope (±2000 °/s) and a 3-triaxial magnetometer (±1.3 125 Gs). The sensors were attached to the lateral aspect of each shoe using special straps and at the level of the fifth lumbar spine segment close to the centre of mass to measure linear acceleration, angular velocity and the magnetic field at a sampling rate of 100 Hz. Step time was defined as the time between two consecutive heel strikes, step time variabilities were calculated extracting standard deviation (SD) from all steps. Asymmetry was defined as the average absolute difference between left and right steps for each walking pass.

Detection algorithm for supervised gait assessment have been already validated in large multicentre studies [1]

**Unsupervised assessment**

Move IV is a three-axial acceleration sensor with a range of ±8 g, a resolution of 12 bit, a sampling rate of 64 Hz and angular rate of 64 degrees/s The recorded acceleration raw data are saved on a micro-SD card. The transmission of raw data after a complete measurement is realized by a USB 2.0 interface. The gait parameters have been extracted and gait parameters assessed as previously reported. [1-2]

***Supplementary Figure 1.*** *Study work flow*


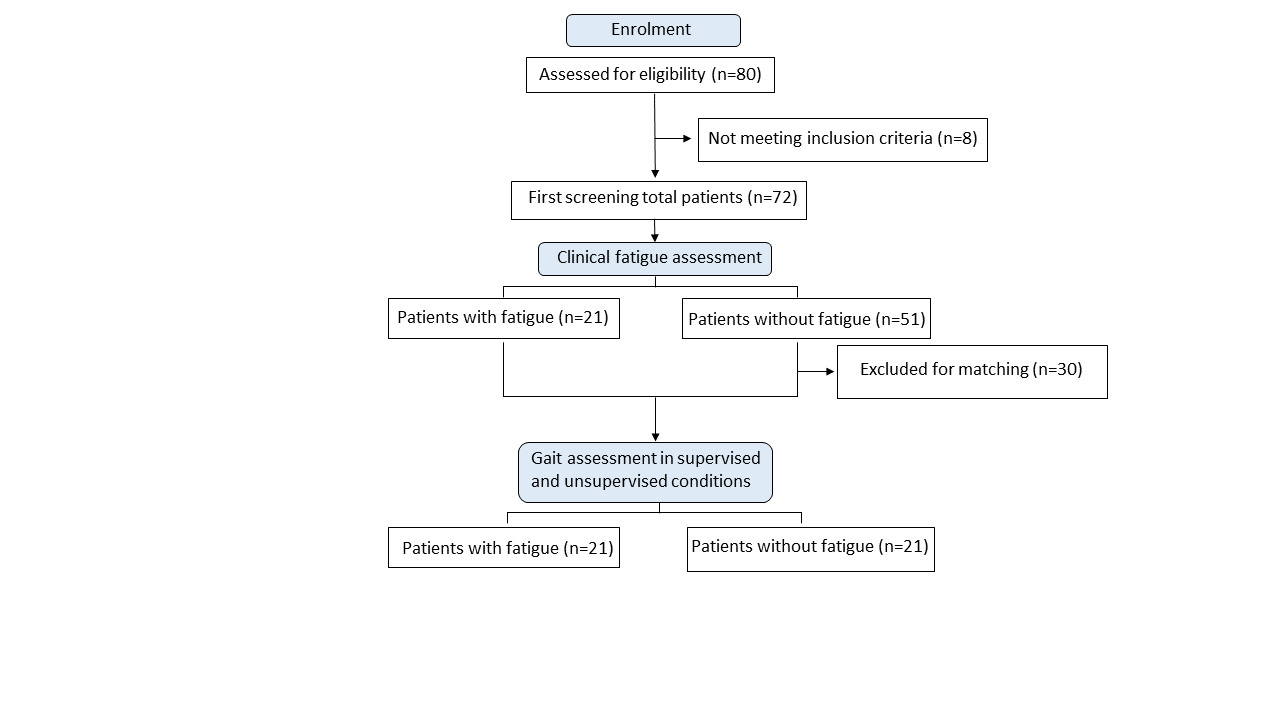


**REFERENCES**

[1] Pham MH, Elshehabi M, Haertner L, Del Din S, Srulijes K, Heger T, Synofzik M, Hobert MA, Faber GS, Hansen C, Salkovic D, Ferreira JJ, Berg D, Sanchez-Ferro Á, van Dieën JH, Becker C, Rochester L, Schmidt G, Maetzler W. Validation of a Step Detection Algorithm during Straight Walking and Turning in Patients with Parkinson's Disease and Older Adults Using an Inertial Measurement Unit at the Lower Back. Front Neurol. 2017 Sep 4;8:457. doi: 10.3389/fneur.2017.00457.

[2] Del Din S, Godfrey A, Rochester L. Validation of an Accelerometer to Quantify a Comprehensive Battery of Gait Characteristics in Healthy Older Adults and Parkinson's Disease: Toward Clinical and at Home Use. IEEE J Biomed Health Inform. 2016 May;20(3):838-847. doi: 10.1109/JBHI.2015.2419317. Epub 2015 Apr 2. PMID: 25850097.
